# Stem Cell Based Therapy Option in COVID-19: Is It Really Promising?

**DOI:** 10.14336/AD.2020.0608

**Published:** 2020-10-01

**Authors:** Duygu Koyuncu Irmak, Hakan Darıcı, Erdal Karaöz

**Affiliations:** ^1^Istinye University, Faculty of Medicine, Department of Histology & Embryology, Istanbul, Turkey; ^2^Istinye University, Stem Cell and Tissue Engineering R&D Center, Istanbul, Turkey; ^3^Istinye University, 3D Bioprinting Design & Prototyping R&D Center, Istanbul, Turkey; ^4^Liv Hospital, Stem Cell and Regenerative Therapies Center (LivMedCell), Istanbul, Turkey

**Keywords:** Stem cell, treatment, COVID-19, clinical, trials

## Abstract

The COVID-19 patients were first detected in China, in December 2019, then the novel virus with associated pneumonia and other diseases spread quickly to worldwide becoming a serious public health intimidation. Despite all the efforts, the pharmacological agents used for controlling or treating the disease, especially respiratory problems, have not been accomplished so far. Among various treatment options, mesenchymal stem cell-based cellular therapies are being investigated, because of their regeneration ability and multipotency along with other features like immunomodulation, antifibrosis and anti-inflammatory effects. This paper intends to analyze the current clinical trials on stem cell treatment of novel virus, searching and reviewing the available information and the International Clinical Trials Registry Platform (ICTRP) of World Health Organization (WHO). We concluded that the stem cell treatment of COVID-19 is found promising with pilot studies’ results, but still in the early development phase. There is an urgent need for large-scale investigations to confirm and validate the safety and efficacy profile of these therapies with reliable scientific evidence.

It has been more than a century, since 1918 in?uenza pandemic unsettled thousands. At the beginning of 2020, the world suddenly faced another pandemic, with a new coronavirus outbreak which is still spreading all over the world, running over all the borders.

World Health Organization (WHO) named the novel virus as SARS-CoV-2 on February 11, 2020, which also called as, 2019 novel coronavirus (2019-nCoV) and the associated disease as Coronavirus Disease-19 (COVID-19) [1].

The warning of WHO emphasized that COVID-19 is a global health emergency, public enemy number 1 and potentially more powerful than terrorism (NEWS A: WHO warns coronavirus, now dubbed COVID-19, is 'public enemy number 1' and potentially more powerful than terrorism’. Broadcasted on Feb 12 2020 in the internet; accessible at: www.abc.net.au/news/2020-02-12/coronavirus-public-enemy-number-onevaccine/11956446). By the time of this paper is written, the outbreak spread all over the world and near 184 other countries all over the world, and the total COVID-19 infection confirmed cases are 2,567,327, total death is 177,521, and total of recovered people are 687,456 as reported by Johns Hopkins guides (Coronavirus COVID-19 Global Cases Dashboard by Johns Hopkins CSSE; www.hopkinsguides.com/hopkins/view/Johns_Hopkins_ABX_Guide/540747/all/Coronavirus_COVID_19__SARS_CoV_2_).

One of the well-known facts about SARS-CoV infection is that it leads the Acute Respiratory Distress Syndrome (ARDS) in severe cases [2]. In some patients, ARDS induces severe pneumonia, and in more severe cases the Multi Organ Dysfunction (MOD) which is currently causing panic all over the world [3]. Despite all the efforts, the pharmacological agents used for controlling or treating the disease, especially curing the respiratory problems, have not been fully successful so far. Among various treatment options, mesenchymal stem cell (MSC)-based cellular therapies are being investigated, because of MSC’s regeneration ability though limitless self-renewal and multipotency along with other features like immunomodulation, antifibrosis and anti-inflammatory effects [4, 5, 6]. Notwithstanding, there has not been a certain consensus on MSC therapy reliability in treating the SARS-CoV-2 induced ARDS or pneumonia in the scientific community.

The aim of this paper is, taking the SARS-CoV-2 infection and stem cell treatment approaches together as main focus all over the world:

1 -to review the published clinical trials information and
2 -to analyze the registered clinical trials information in International Clinical Trials Registry Platform (ICTRP) of WHO and the partner registries providing to this joint registry
3 -to interpret on the strengths and hurdles of the stem cell treatment option in SARS-CoV-2 infected patients under the light of the available scientific evidence.

## Etiopathogenesis of the SARS-CoV-2 Infection: Breeze or Storm of Cytokines

The CoV subfamily is composed of large, enveloped viruses with a single strand of sense RNA. Among these, the alpha and beta-CoVs are known to infect humans [7, 8]. While they usually cause mild respiratory infection symptoms, the first coronavirus epidemic in 2002 by SARS-CoV and in 2012 by Middle East respiratory syndrome coronavirus (MERS-CoV) lead to severe and even fatal symptoms [9].

Coronavirus, named after the spike-like glycoproteins (S proteins) surrounding the viral envelope like a crown [10]. These S proteins binds the virus to its cellular receptors, angiotensin-converting enzyme 2 (ACE2) in SARS-CoV and dipeptidyl peptidase 4 (DPP4) in MERS-CoV, with subsequent membrane fusion [11]. Cell cytoplasm meets the viral RNA genome, then the viral genome is replicated. The virion-containing vesicles consists of genomic RNA, envelope glycoproteins and nucleocapsid proteins develops, then fuses with the plasma membrane, resulting in the release of the viruses [12]. SARS-CoV-2, was found to be a new type of beta-CoV with more than 99.98% genetic identity similarity to SARS-like coronaviruses and was reported as genetically more identical to SARS-CoV than to MERS-CoV [13, 14].

Another reported structural aspect of SARS-CoV-2 is that its ability to form a novel short protein encoded by orf3b region. It has been suggested that the orf3b of SARS-CoV-2 possibly play a role in the viral pathogenicity and inhibit the expression of IFNβ [15].

The expression of ACE2 is most abundant in endothelial cells and smooth muscle cells in the organs, facilitates the spreading of the virus as it enters the blood circulation. Also, since the ACE2 receptor is quite common in cell membrane, they especially exist particularly in the Alveolar Type 2 progenitor (AT2) cells in the lungs as well as in the kidneys, heart, liver, and digestive organs [16, 17, 18]. All tissues and organs expressing ACE2 receptors are the targets of the SARS-CoV and the fighting immune cells. Consequently, besides ARDS, caused by the damaged lung cells, the infected patients might also experience acute myocardial injury, arrhythmia, acute kidney injury, shock, and death from multiple organ failure [19, 20].

Inflammation is of crucial importance for effective immune response in order to succeed in resolution of infection and cell damages. The in?ammatory response, here we may use breeze term, is responsible for initial recognition of an infection, attracting the cells in charge to allow resolution. Hence the breeze is made by the inflammatory cells secretions which are many cytokines to serve to homeostasis [21]. If the immune system is over activated due to the viral overload, a quite large number of inflammatory factors are secreted, and severe cytokine storm develops [19-23]. IL-2, IL-6, IL-7, GSCF, IP10, MCP1, MIP1A, and TNFα are the factors mainly causing the cytokine storm. Later, edema and then ARDS develops. If secondary infections make the cases much more severe [20].

As this new viral pandemic is totally new for human being, currently science community learns about the immunity and susceptibility of the population, taking the knowledge from the previous studies on other coronavirus infections as base and combining all reported new cases and research outcomes.

SARS- CoV-2 might interfere the immune pathways at least in two levels: as innate and adaptive immunity responses (Fig. 1). When the virus invades the host, the pattern recognition receptors (PRRs), Toll-like receptors (TLR), NOD-like receptors (NLR), and RIG-I- like receptors (RLR) work on the recognition of the virus by innate immune system. The virus promotes dendritic cells maturation, expression of the inflammatory factors, and type I interferons (IFNs) release. These induce macrophage function and thus viral invasion restriction [24]. SARS- CoV’s N protein can induce the interference of the immune process by supporting the virus run off the immune responses [25]. As parts of the adaptive immune system, the main actors, CD4+ T cells work by stimulating B cells to produce virus-specific antibodies, while CD8+ T cells directly kill virus-infected cells. On the other hand, T helper cells secrete the proinflammatory cytokines to support the defense of the body. Anyhow, nCoV is able to suppress the function of T cells by inducing their apoptosis. On the other hand, if the immune system is overreacted, local reactive free radicals may provoke severe damages to the organs, particularly to the lungs [26].

**Figure 1.**
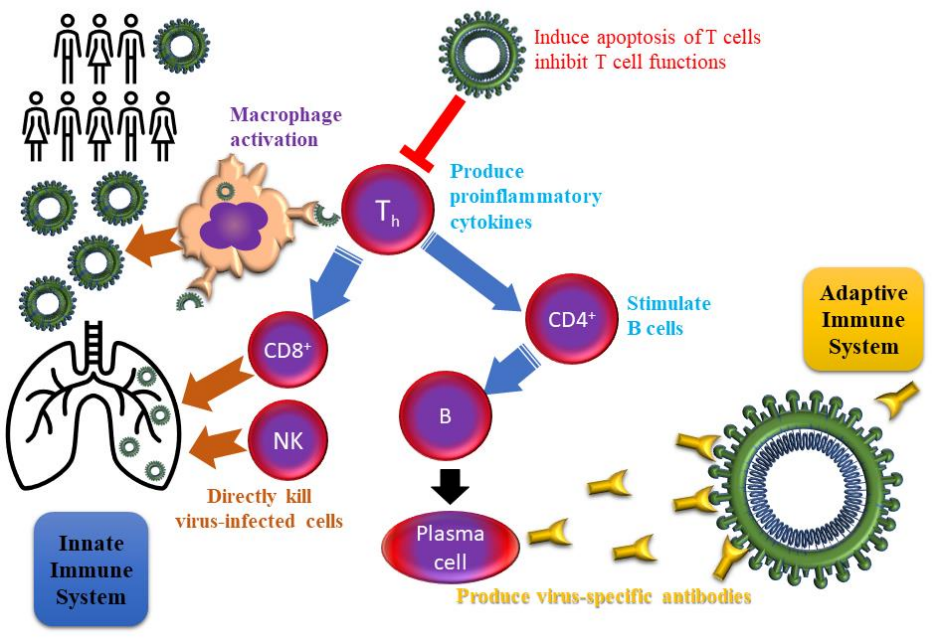
Demonstration of the SARS-CoV impact during the general immune reaction function. (PRRs: pattern recognition receptors, TLR: Toll-like receptor, NLR: NOD)-like receptors, RLR: retinoic acid-inducible gene-I (RIG-I)-like receptors, IFN: Interferone).

## Stem Cells as An Option Curing SARS-CoV Induced Pulmonary Impairment

Regenerative medicine and tissue engineering are the most exciting cell-based therapeutic applications [27]. In these approaches, the cells are utilized for replacing or rebuilding the damaged organs and tissues [28, 29]. At the beginning stages of the cellular therapies, the methods of administering the cells into the injured tissue areas, such as skeletal myocytes injected into post-myocardial infarction scar tissue or neuronal cells into the brain of patients with Parkinson’s disease [30, 31]. The other methodologies are using the extracorporeal organ replacement for acute renal failure or fulminant acute liver failure, skeletal stem cell implantation for bone regeneration, which are already under clinical application [33, 34]. As the considerable amount of evidence has been obtained from basic research and its translation towards the clinical research, MSCs placed in medical practice as an alternative treatment option as safe and effective cell-based therapies for immune-mediated inflammatory diseases such as graft versus-host disease (GVHD) and systemic lupus erythematosus (SLE) [34-37].

Obviously, stem cell treatment has not yet been studied in a detailed manner in wide range of patients in multinational well-designed studies in Acute Respiratory Distress Syndrome or pneumonia cases caused by SARS-CoV, except for some pilot studies.

MSCs have been demonstrated to improve organ functions in cardiovascular, renal, hepatic, and multiple other disorders. Another function of stem cell is their ability to regulate the inflammatory response and promote tissue repair and regeneration. MSCs perform their therapeutic effects mainly in two ways:

1 -Proliferation and differentiation abilities for organ regeneration upon damage.
2 -immunomodulatory effects via
  a. The paracrine secretion of many cytokine types,
  b. Conducting messages via crosstalk with immune cells including T cells, B cells, dendritic cells, macrophages and natural killer cells leading to immunomodulation
  c. Through extracellular vesicles, mainly exosomes.

One suggested function of stem cells is their ability to regulate the inflammatory response and promote tissue repair and regeneration. MSCs have been demonstrated to improve organ functions in cardiovascular, renal, hepatic, and multiple other disorders [34-37].

Attractivity of the MSCs as treatment agents, in addition to their cellular characteristics mentioned above, is coming from their wide range of sources. MSCs can be isolated from different adult and neonatal tissues. Adult bone marrow, peripheral blood (PB) and various adipose tissues (AT) can be used as roots, whereas umbilical cord blood, Warton jelly and cord itself, amniotic fluid, placenta can be the neonatal sources of these cells. Their low-invasive usability of MSCs in the clinics is also a promising feature of the treatment. Also, menstrual blood-derived MSC is attracting interest in experimental efforts [38-41].

There have been some evidence suggesting that the MSC therapy has considerable superiority when compared with other treatments in for example, skin diseases [42].

Evidence also suggests that the umbilical cord derived MSC exosomes which are extracellular vesicles of 40-100nm in size, have the same functionality as MSCs, with some superiority MSCs injection complications [43]. MSC exosomes show CD9, CD63, CD81 markers expression, and contain some cytokines including high levels of IL-6 and IL-8. These exosomes stimulate cell proliferation and protection against H2O2 induced apoptosis [44] also reported as having no harmful e?ects on the kidneys and liver. Some studies present evidence on as systemic anaphylaxis, hemolysis, pyrogen, hematology indexes, vascular and muscle stimulation suggest that the exosomes maybe evaluated as safe therapeutic mediators [45]. Some other evidence also reported by a number of investigators in regards to the exosome therapy in kidney diseases [46, 47], ocular disease [48, 49], Alzheimer’s disease [50, 51], spinal cord injury [52, 53] and acute myocardial ischemia [54, 55].

When it comes to finding effective and safe cures for the patients suffering from COVID-19, the research hypotheses were set generally around the idea that MSCs as treatment agents can be effective to prevent the cytokines’ storm over the immune system and to promote tissue repair endogenously by reparative capabilities of these cells. When infused in intravenous route, MSCs joins to the pulmonary tissue and induce the recovery of the pulmonary cellular level impairment and treat lung dysfunction and COVID-19 pneumonia [19, 56].

## Clinical Trials Registries as Source of Key Investigational Information

All stakeholders having involved in all stages of the clinical trials, such as doctors, scientists, industry, academics, and most importantly the patients, share a common right and eagerness for a vigorous clinical research enterprise that brings innovations to all parties as quickly and as transparently as possible. The transparency in clinical research involves registration of clinical trials on public databases, accessibility of patient data for subsequent analysis, and publication of results irrespective of the trial outcome. The publicly available clinical trial data over registries keeps all parties involved in the clinical trials up to date on the recent medical research trends and results even before they are published. It is of crucial importance to have transparent results to ensure that decisions related to the safety and efficacy of investigated treatment or prevention approaches to be used in human are supported by the best-available scientific evidence. Evidence-based public health decisions and strategies ensure, in turn, an optimal allocation of public health resources and, ideally, better patient outcomes (https://www.ecrin.org/node/583 access 24 April 2020).

International Clinical Trials Registry Platform (ICTRP) is an initiative of World Health Organization (WHO) established with the purpose of ensuring the accessibility of clinical research by all involved parties. Thus, the initiative is serving for improving not only the transparency but also the validity and reliability of the trials in means of scientific evidence worldwide (www.who.int/ictrp/en/ access 18 April 2020).

WHO Registry, ICTRP, is maintained by a network meeting the specific criteria for content, quality and validity, accessibility, unique identification, technical capacity and administration International Committee of Medical Journal Editors (ICMJE). The partners and data providers of the ICTRP are listed Table 1 with the web sites.

**Table 1:** Clinical trials and web sites.

	Registry of Clinical trials	Web Site
1	Australian New Zealand Clinical Trials Registry (ANZCTR)	http://www.anzctr.org.au/
2	Brazilian Clinical Trials Registry (ReBec)	http://www.ensaiosclinicos.gov.br/
3	Chinese Clinical Trial Registry (ChiCTR)	http://www.chictr.org.cn/index.aspx
4	Clinical Research Information Service (CRiS), Republic of Korea	http://ctri.nic.in/Clinicaltrials/login.php
5	United States National Library of Medicine	https://www.clinicaltrials.gov
6	Clinical Trials Registry - India (CTRI)	http://ctri.nic.in/Clinicaltrials/login.php
7	Cuban Public Registry of Clinical Trials (RPCEC)	http://registroclinico.sld.cu/en/home
8	EU Clinical Trials Register (EU-CTR)	https://www.clinicaltrialsregister.eu/
9	German Clinical Trials Register (DRKS)	https://www.drks.de/drks_web/
10	Iranian Registry of Clinical Trials (IRCT)	https://www.irct.ir/
11	Japan Primary Registries Network (JPRN)	https://rctportal.niph.go.jp/en/
12	Thai Clinical Trials Registry (TCTR)	http://www.clinicaltrials.in.th/
13	Lebanese Clinical Trials Registry (LBCTR)	http://lbctr.emro.who.int/Trials/View#
14	The Netherlands National Trial Register (NTR)	https://www.trialregister.nl/
15	Pan African Clinical Trial Registry (PACTR)	https://pactr.samrc.ac.za/
16	Peruvian Clinical Trial Registry (REPEC)	https://ensayosclinicos-repec.ins.gob.pe/en/
17	Sri Lanka Clinical Trials Registry (SLCTR)	http://www.slctr.lk/

The COVID-19 pandemic has alerted the WHO, in a number of areas, including public health warnings, preventive actions recommendations, and follow up the cases’ data to release. Among these, the ICTRP registry of clinical trials become one of the major sources to track the clinical trials investigating the safe and effective cure’s for COVID-19 catastrophe.

## Method and Analysis

The focus of this paper is reviewing the published and unpublished information about the clinical trials having the stem cell therapy on COVID-19. For this purpose, the published literature search was made in the PubMed and Google Scholar, and the registry searching of the clinical trials is done through the ICTRP where 17 registry data bases have been contributing in (Table-1).

The key words “COVID-19 OR Coronavirus OR New Coronavirus OR SARS-CoV-19 AND stem cell AND Treatment” were used in all levels. The date of search is 18 April 2020. The clinical research, regardless of being published or not, are selected where the phase of the trials are mentioned clearly, so all observational, basic sciences, diagnostic test, epidemiologic research, expanded access, health services research, prevention, prognosis, and screening trials are excluded whereas the interventional and treatment trials which are active recruiting or not yet recruiting are selected.

A further selection is done for trials mainly on stem cell treatment, so the trials with other interventions are excluded. The trial selection in ICTRP database is demonstrated in Figure 2.

The analysis of the data is done to find out the trend of the trials’ treatment targets over stem cell usage, the participant selection preferences, eligibility and primary trial outcome planning along with the methodology of the trials.

## Findings

Total 16 trials registered in ICTP are selected for review and analysis. The trials with titles and identification numbers (ID) are listed in Table 2. This table also shows the enrolment status and the age range defined per trial.

Majority of the studies were actively recruiting (11), while some of the studies (5) were not yet recruiting so far.

**Figure 1.**
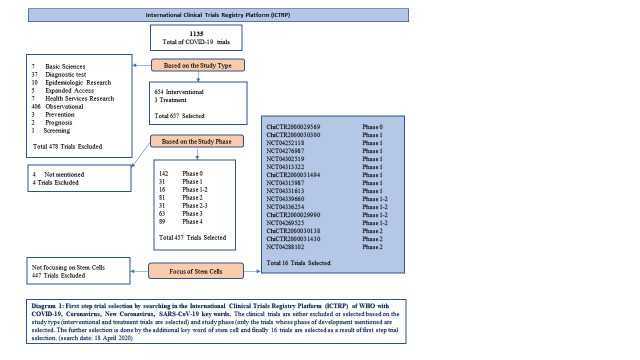
First step trial selection by searching in the International Clinical Trials Registry Platform (ICTRP) of WHO with COVID-19, Coronavirus, New Coronavirus, SARS-CoV-19 key words. The clinical trials are either excluded or selected based on the study type (interventional and treatment trials are selected) and study phase (only the trials whose phase of development mentioned are selected. The further selection is done by the additional key word of stem cell and finally 16 trials are selected as a result of first step trial selection. (search date: 18 April 2020)

## Phases/Indication/Condition

We specified that all trials selected are of early development phases; there were no phase 3 or 4 studies in the registries.

*Early phase 1* (formerly called phase 0) which is early exploratory study phase was the development stage of one randomized controlled non-blinded trial (ChiCTR2000029569).

*Phase 1* (First in human safety) design was used in one parallel controlled (ChiCTR2000029569), one parallel open label (NCT04252118), one single arm (NCT04276987), and three single arm open label (NCT04313322, NCT04315987, NCT04302519) trials.

*Phase 1-2* (Safety and Therapeutic Confirmatory) design was the core of two randomized controlled triple blinded trials (NCT04339660, NCT04336254); one parallel controlled trial (ChiCTR2000029990), and one single arm open label trial (NCT04331613).

*Phase 2* (Therapeutic Confirmatory) trials were one randomized controlled Quadruple ((Participant, Care Provider, Investigator, Outcomes Assessor) -blinded (NCT04288102), one parallel controlled (ChiCTR2000031430), and one single arm open label (NCT04269525) trials.

Almost all of the trials selected have the common purpose of treating the SARS-CoV related pneumonia or Acute Respiratory Distress Syndrome.

## Participants/patients perspective

The age range selected in the trials was non-paediatric subjects for all studies and reaching outcome measures in the adult population is targeted. Only one trials shows the minimum age rage as 16 years (ChiCTR2000030138), but rest takes the smallest age as 18. Maximum recruitment age was not defined in 3 trials, average of the remaining studies’ maximum age is 76.7.

**Table 2:** Selected trials in ICTP of WHO.

Trial ID	Public title	Recruitment Status	Inclusion age	
			min	max
ChiCTR2000029569	Safety and efficacy of umbilical cord blood mononuclear cells conditioned medium in the treatment of severe and critically novel coronavirus pneumonia (COVID-19): a randomized controlled trial	Not Recruiting	18	-
ChiCTR2000030138	Clinical Trial for Human Mesenchymal Stem Cells in the Treatment of Severe Novel Coronavirus Pneumonia (COVID-19)	Not Recruiting	16	75
ChiCTR2000029990	Clinical trials of mesenchymal stem cells for the treatment of pneumonitis caused by novel coronavirus pneumonia (COVID-19)	Recruiting	18	95
ChiCTR2000030300	Umbilical cord mesenchymal stem cells (hucMSCs) in the treatment of high-risk novel coronavirus pneumonia (COVID-19) patients	Recruiting	18	75
ChiCTR2000031494	Clinical study for stem cells in the treatment of severe novel coronavirus pneumonia (COVID-19)	Recruiting	18	90
ChiCTR2000031430	Clinical study of human umbilical cord mesenchymal stem cells in the treatment of novel coronavirus pneumonia (COVID-19) induced pulmonary fibrosis	Recruiting	18	80
NCT04252118	Mesenchymal Stem Cell Treatment for Pneumonia Patients Infected With COVID-19	Recruiting	18	70
NCT04276987	A Pilot Clinical Study on Inhalation of Mesenchymal Stem Cells Exosomes Treating Severe Novel Coronavirus Pneumonia	Not yet Recruiting	18	75
NCT04313322	Treatment of COVID-19 Patients Using Wharton’s Jelly-Mesenchymal Stem Cells	Recruiting	18	-
NCT04315987	NestCell® Mesenchymal Stem Cell to Treat Patients With Severe COVID-19 Pneumonia (HOPE)	Not yet Recruiting	18	-
NCT04331613	Safety and Efficacy of CAStem for Severe COVID-19 Associated With/Without ARDS	Recruiting	18	70
NCT04339660	Clinical Research of Human Mesenchymal Stem Cells in the Treatment of COVID-19 Pneumonia	Recruiting	18	75
NCT04336254	Safety and Efficacy Study of Allogeneic Human Dental Pulp Mesenchymal Stem Cells to Treat Severe COVID-19 Patients	Recruiting	18	65
NCT04288102	Treatment With Mesenchymal Stem Cells for Severe Corona Virus Disease 2019 (COVID-19)	Recruiting	18	75
NCT04302519	Novel Coronavirus Induced Severe Pneumonia Treated by Dental Pulp Mesenchymal Stem Cells	Not yet Recruiting	18	75
NCT04269525	Umbilical Cord (UC)-Derived Mesenchymal Stem Cells (MSCs) Treatment for the 2019-novel Coronavirus (nCOV) Pneumonia	Recruiting	18	75

The gender is defined as both genders at all trials, except for one trial (ChiCTR2000030138).

## Trial Methodology

The protocol design, development phases, participant size blinding status and the treatment intervention types to cure SARS-CoV infection of the randomized controlled trials are summarised in Table 3. There were 5 randomized controlled studies which are 1 non blinded (ChiCTR2000029569), 1 double blinded (ChiCTR 2000030138), 2 triple-blinded (NCT04339660, NCT 04336254) and 1 quadruple-blinded (NCT04288102) trials identified. (Table 3). All trials in this group combine the conventional or routine treatment with multiple MSCs’ infusions in varying doses, except for one trial where the routine treatment combination is mentioned only in control group not in the experimental group (ChiCTR2000030138).

The rest of the trials are 3 Parallel controlled studies (ChiCTR2000029990, ChiCTR2000031494, ChiCTR 2000031430), (Table 4) 1 parallel Open label study (NCT04252118), 1 Single arm trial (NCT04276987), and 5 single arm open label trials (NCT04313322, NCT04315987, NCT04331613, NCT04302519, NCT 04269525) (Table 5).

1 Case Series trial (ChiCTR2000030300) was cancelled during this clinical trials’ analysis with justification of “Cancelled by the investigator Umbilical cord mesenchymal stem cells (hucMSCs) in the treatment of high-risk novel coronavirus pneumonia (COVID-19) patients”.

**Table 3:** The randomised controlled trials along with their development phases, participant size blinding status and the treatment intervention types to cure SARS-CoV infection in ICTP database of WHO.

Protocol design: Selected Trials with Randomized Controlled Design			
Trial ID	Phase / Target participants	Blinding	Intervention
ChiCTR2000029569	Phase 0 / 30 subjects	Open label	Experimental group: conventional treatment combined with *umbilical cord mesenchymal stem cell*conditioned medium group Control group: Conventional treatment
NCT04339660	Phase 1-2 / 30 Subjects	Triple-blinded	Experimental group: conventional and treatment with *umbilical cord MSCs*, 1x10^6^ UC-MSCs /kg body weight suspended in 100mL saline Control group: conventional treatment and Placebo intravenously. Placebo 100mL saline intravenously
NCT04336254	Phase 1-2 / 20 Subjects	Triple-blinded	Experimental group: *allogeneic human dental pulp stem cells* Intravenous injection of 3.0x10^7^ human dental pulp stem cells solution (30ml) on day 1, day 4 and day 7, based on routine treatment of COVID-19 Control group: Intravenous saline injection as Placebo Intravenous injection of 3ml of 0.9% saline on day 1, day 4 and day 7, based on routine treatment of COVID-19
NCT04288102	Phase 2 / 90 Subjects	Quadruple-blinded	Experimental Group: Conventional treatment plus 3 times of MSCs(4.0x10^7^ cells per time) intravenously at Day 0, Day 3, Day 6. Control Group: Saline containing 1% Human serum albumin (solution of MSC) 3 times of placebo (intravenously at Day 0, Day 3, Day 6)Other: Intravenous saline injection (Placebo) Intravenous injection of 3ml of 0.9% saline on day 1, day 4 and day 7, based on routine treatment of COVID-19
ChiCTR2000030138	Phase 2 / 60 subjects	Double Blinded	Experimental group: Intravenous injection of *human umbilical cord mesenchymal stem cells (UC-MSC*); *Note: routine treatment combination was not mentioned in the other records.* Control Group: Routine treatment + placebo

## Intervention

In all selected trials selected, the mesenchymal stem cells (MSCs) are used as biological treatment agent. The sources of the MSCs used in the trials selected as follow:

1 -Umbilical cord
2 -Wharton Jelly
3 -Human dental pulp
4 -Embryonic origin

In one trial MSCs-derived exosomes are used. We also identified one trial using commercially manufactured MSC, namely NestCell®.

All forms of the MSCs are planned to given to the patients as intravenous infusion, except one trial (NCT04276987) in which the exosomes of MSCs are given by inhalation; as single to multiple applications with one- or two-days intervals up to 21-28 days (Table 6).

**Table 4:** The Parallel Controlled Design along with their development phases, participant size blinding status and the treatment intervention types to cure SARS-CoV infection in ICTP database of WHO.

Protocol design: Selected Trials with Parallel Controlled Design			
Trial ID	Phase	Total target Participants	Intervention
ChiCTR2000029990	Phase 1-2	120 Subjects	Experimental group: mesenchymal stem cells. Control group: saline. *Note: Randomly divided into group A as placebo group and group B as stem cell treatment group*
ChiCTR2000031494	Phase 1	36 Subjects	Experimental group: Conventional medication + Infusion of mesenchymal stem cells; Control group: Conventional medication.
ChiCTR2000031430	Phase 2	200 Subjects	Experimental group: Conventional treatment regimen + MSC treatment; Control group: Conventional treatment regimen.

**Table 5:** The trials with other design characteristics along with their development phases, participant size blinding status and the treatment intervention types to cure SARS-CoV infection in ICTP database of WHO.

Protocol design: Selected Trials with Other Design Characteristics			
Trial ID	Phase / Target Participants	Design	Intervention
NCT04252118	Phase 1 / 20 Subjects	parallel open label	Experimental Group: 3 times of MSCs (3.0*10E7 MSCs intravenously at Day 0, Day 3, Day 6). Control Group: Without MSCs Therapy but conventional treatment should be received.
NCT04276987	Phase 1 / 30 Subjects	Single arm	*MSCs-derived exosomes* (5 times aerosol inhalation of MSCs-derived exosomes (2.0*10E8 nano vesicles/3 ml at Day 1, Day 2, Day 3, Day 4, Day 5).)
NCT04313322	Phase 1 / 5 Subjects	Single arm open label	*WJ-MSCs* will be derived from cord tissue of newborns, screened for HIV1/2, HBV, HCV, CMV, Mycoplasma, and cultured to enrich for MSCs.
NCT04315987	Phase 1 / 66 Subjects	single arm open label	*NestCell®* is a mesenchymal stem cell therapy produced by Cellavita
NCT04331613	Phase 1-2 / 9 Subjects	single arm open label	CAStem will be administered intravenously. *Human Embryonic Stem Cells Derived M Cells (CAStem)* A dose-escalation with 3 cohorts with 3 patients/cohort who receive doses of 3, 5 or 10 million cells/kg. If there are no safety concerns for each cohort, the dose will be escalated from lower dose to next higher dose.
NCT04302519	Phase early 1 / 24 Subjects	single arm open label	*Dental pulp mesenchymal stem cells* On the basis of clinical standard treatment, the injection of dental mesenchymal stem cells was increased on day 1, 3 and 7 of the trial.

## Primary Study Objectives and Outcome:

We found out that the selected clinical trials have the clinical trial objectives and trial outcomes defined in conjunction to the objectives. The study outcomes are defined as primary and secondary ones, as it is the case in the study objectives. Taking the fact that a sensible strategy in a study is to establish a single primary research question around which to focus the study plan, we evaluated the study objectives versus primary study outcomes listed in the registry in this paper.

**ChiCTR2000029569**: This study’s target patient population is *severe and critical* 2019-ncov coronavirus pneumonia. The main objective is to determine the *effectiveness and safety* of the conventional treatment versus conventional plus UC-MSC treatment. The study has the outcome measure Pulmonary Severity Index (PSI) to get the data.

**ChiCTR2000030138**: This trial is conducted to evaluate the *effectiveness and safety* of UC-MSCs in the treatment of 2019-nCoV infected pneumonia patients. In this trial the severity of the disease state is not specified. The outcome measure is taken as clinical index for obtaining outcome.

**ChiCTR2000029990**: This is also an *effectiveness and safety* study having target cases as treatment of new coronavirus pneumonitis not specifying the severity of the clinical appearance. Improved respiratory system function and recovery time as demonstrated by the level of blood oxygen saturation is the outcome measure.

**ChiCTR2000030300**: (Cancelled study during this paper’s analysis) The trial has the objective of evaluating the *clinical effect of* UC-MSCs in high risk of novel coronavirus pneumonia patients to support treatment and to improve the pulmonary fibrosis in the later stage. The safety is not listed as objective. The outcome measures are listed as time to disease recovery, time and rate of coronavirus become negative, and exacerbation (transfer to RICU) time;". This trial’s cancellation reason was not declared in the registry.

ChiCTR2000031494: This is another trial having objective of evaluating if the UC-MSCs have *efficacy and more benefits* in treating Corona Virus Disease 2019. As the outcome points are listed as chest imaging, lung function, and ADL, it is understood that the outcome measures are simply of pulmonary status. However, the clinical appearance of the disease, or any severity definition is not included in the study records.

ChiCTR2000031430: Evaluating the *efficacy and safety* of UC-MSCs in COVID-19 induced pulmonary fibrosis treatment. Electrocardiogram, St George’s Respiratory Questionnaire, Score, high resolution CT for chest, blood gas analysis, percutaneous blood oxygen saturation, 6 min walking distance, pulmonary function VCmax, blood routine, liver and kidney function, cytokine analysis, immunoglobulin, lymphocyte subsets, coagulation, myocardial enzymes, serum ferritin, procalcitonin (PCT), IL-6, lactic acid, D-Dimer, CRP are chosen as outcome measure showing that the trial’s expectation is to understand how the stem cell therapy make its impact in tissue level as indicated cytokines and parameters, also to find out if this therapy has a systematical adverse effect resulting in the multi organ biochemistry data changes.

**Table 6:** The MSC treatment doses, route of administration, treatment/ application intervals and target lung condition’s name and grade (mild, moderate, severe or critical).

Trial ID	Therapy	Route	Dose	Application intervals	Condition
ChiCTR2000029569	Conventional treatment (CT) combined with UC-MSC conditioned medium	Not mentioned	Not mentioned	Not Mentioned	Severe or/to critical pneumonia diagnosis due to SARS-CoV infection
ChiCTR2000030138	CT + UC-MSC CT + placebo	IV injection	Not mentioned	Not Mentioned	Severe Novel Coronavirus Pneumonia
ChiCTR2000029990	MSCs or saline	Not mentioned	Not mentioned	Not Mentioned	Moderate to severe cases of new coronavirus pneumonia
ChiCTR2000030300 (Cancelled)	(UC-MSCs)	Not mentioned	Not mentioned	Not Mentioned	Patients with high risk
ChiCTR2000031494	CT or CT plus UC-MSCs	IV	Not mentioned	Not Mentioned	Severe novel coronavirus pneumonia
ChiCTR2000031430	CT + UC-MSCs	IV	4*10^7^ cells/application	on days 0, 3, and 6 for a total of 3 times	Novel Coronavirus Pneumonia (COVID-19) severity not mentioned
NCT04252118	CT plus MSCs	IV	1*10^7^cells/application	at Day 0, Day 3, Day 6).	Pneumonia Patients Infected With COVID-19 severity not mentioned
NCT04276987	CT plus MSCs-derived exosomes	aerosol inhalation	1*10^8^ nano vesicles/3 ml	at Day 1, Day 2, Day 3, Day 4, Day 5	Severe or/to critical pneumonia diagnosis due to SARS-CoV infection
NCT04313322	WJ-MSCs	IV	1*10^6^ cells/kg	The three doses will be 3 days apart form each other. 3 weeks follow up	COVID-19 (ever, respiratory destress, pneumonia, cough, sneezing, diarrhea) severity not mentioned
NCT04313322	WJ-MSCs	IV	1*10e^6^ cells/kg	The three doses will be 3 days apart form each other.	COVID-19 (ever, respiratory destress, pneumonia, cough, sneezing, diarrhea) severity not mentioned.
NCT04315987	CT plus MSCs	IV	2*10^7^cells/application	on days 1, 3 and 5 and if necessary on day 7	Severe COVID-19 pneumonia.
NCT04331613	CAStem, immunity- and matrix-regulatory cells (IMRCs), differentiated from hESCs	IV	3/5/10*10^6^ cells/kg. Escalating doses.	intervals not mentioned	3 cohorts with 3 patients/cohort with severe COVID-19 associated w/wo ARDS.
NCT04339660	CT plus MSCs	IV	1*10^6^ cells/kg	1 time, or with an interval of 1 week	Critically ill COVID-19 pneumonia
NCT04336254	CT plus hDP-MSCs	IV	3*10^7^cells/bag * 3 bags	injection of on day 1, day 4 and day 7	Severe pneumonia caused by COVID-19
NCT04288102	CT plus MSCs	IV	4.0*10^7^	at Day 0, Day 3, Day 6.	COVID-19 Patients with Severe Convalescence
NCT04302519	CT plus hDP-MSCs	IV	1.0x10^6^ cells /kg.	on day 1, 3 and 7 of the trial.	Novel Coronavirus Induced Severe Pneumonia
NCT04269525	UC-MSCs	IV	3.3*10^7^/50ml bags*3bag,	on day 1, day 3, day 5, and day 7.	Serious Pneumonia and Critical Pneumonia Caused by the 2019-nCOV Infection

NCT04252118: The *safety and efficiency* trial of MSCs treatment in pneumonia patients infected with SARS-CoV-2, is conducted to monitor the pneumonia improvement and side effects profile in certain times within 28 days of treatment. The adverse event monitoring is planned to continue as 6 months. The severity of pneumonia is not defined.

NCT04276987: This pilot trial is aimed to explore the *safety and efficiency* of using aerosol inhalation of the non-cellular exosomes derived from allogenic adipose MSCs as therapy agent in severe patients with novel coronavirus pneumonia. Both efficiency and the safety are planned to be followed up to 28 days. The severity of pneumonia is not defined.

NCT04313322: This trial is conducted to investigate the *potential effective use* of Wharton’s Jelly MSCs for treatment of patient diagnosed with Corona Virus SARS-CoV-2 infection and showing symptoms of COVID-19. The clinical outcome is defined as improvement in clinical symptoms, improvement in pulmonary impairment as demonstrated by CT-Scan and the potential side effects by radiography, also the Real-Time PCR of Viral RNA positivity in 3 weeks. The severity of the disease is not defined.

NCT04315987: This trial is to assess the *efficacy*of commercial product NestCell® as an add-on therapy to standard treatment to treat patients with severe COVID-19 pneumonia. The outcome measure is taken as the change in the clinical condition in 10 days. Collecting the safety is not among the main objectives of the trial.

NCT04331613: In this dose escalation, *safety and early efficacy* study, the CAStem product which is product composed of immunity- and matrix-regulatory cells (IMRCs) (M cells) differentiated from clinical-grade human embryonic stem cell is used. The trial is designed to use CAStem to treat severe COVID-19 associated with or without ARDS. Both frequency and severity of adverse events and serious adverse events are planned to be collected in 28 days along with changes in pulmonary imaging examinations.

NCT04339660: In order to find a potential *effective* cure alternative especially in critically ill COVID-19 pneumonia patients, this trial is planned as adoptive transfer therapy of MSCs. The outcomes are focused on collection of the immune function data over cytokines, improvement and recovery time of inflammatory and immune factors, blood oxygen saturation, and evaluation of pneumonia change in 4 weeks of treatment.

NCT04336254: This *safety and efficacy* study is of allogeneic human dental pulp MSCs in the treatment trial of severe pneumonia cases caused by COVID-19. The objective is to explore the effects this treatment in terms of reducing mortality, improving clinical prognosis, and so discovering a new therapeutic strategy for COVID-19 using allogeneic human dental pulp MSCs. The primary outcome measure is defined as time to clinical improvement. The pulmonary evaluations, blood tests of immune function, and the adverse effects follow up is listed in secondary outcome measures not in primary ones.

NCT04288102: This clinical trial is designed to inspect the *safety and efficiency* of MSCs therapy in severe COVID-19 cases. Size of lesion area and severity of pulmonary fibrosis by imaging and functional tests, oxygen saturation, oxygenation index, duration of oxygen therapy, side effects, immunological characteristics (immune cells, inflammatory factors, etc.) are planned to be evaluated during the 90 days follow up. The safety outcome measures are listed in secondary outcomes.

NCT04302519: The study is on the evaluation of novel coronavirus induced severe pneumonia by dental pulp MSCs treatment. The study is focusing on the treatment *efficiency* as to be demonstrated by the disappearance time of ground-glass shadow in the lungs in 14 days.

NCT04269525: The trial is dealing with the serious pneumonia and critical pneumonia caused by the 2019-nCOV infection. This study is conducted to find out whether or not UC-MSCs treatment will function in 2019-nCOV infection pneumonia treatment, so this is another *efficiency* study. The outcome measure is defined as oxygenation index in 14 days.

## Discussion

The COVID-19 patients were first detected in Wuhan, China, in December 2019, then the novel virus with associated pneumonia and other diseases spread quickly to worldwide becoming a serious public health intimidation [57, 58].

As experienced in the past, in two other lethal coronaviruses SARS-CoV and MERS-CoV [60], novel virus SARS-CoV-2 over-stimulates the immune reactions in the host with concurrent cytokine storm (CS) which are followed by acute lung injury (ALI)/acute respiratory distress syndrome (ARDS). The clinical case worsens resulting in multiple organ failure and death [21, 60]. Even when the treatment in the intensive care units of CS cases goes well, the long-lasting inflammation causes serious pulmonary fibrosis, which in turn destines to the lung dysfunction and very low life-quality [61].

Currently, there are no specific drugs or vaccines are available to cure the patients with COVID-19 pneumonia, except the efforts for the existing drugs’ repurposing, such as the case of antiviral treatments already in the market, or supporting treatments [22, 62]. There is an enormous unmet medical need for a safe and effective treatment for COVID-19 pneumonia patients, associated systemic diseases, especially the severe or critically ill cases.

In this context, the MSCs and related cell-based therapy is considered as a promising option. the MSCs have been widely studied in basic research and clinical trials and these have been studied in a number of diseases [63, 64]. The published evidence reveals the safety and effectiveness of this therapeutic approach in a number of clinical trials, particularly in the immune-mediated inflammatory diseases, such as GVHD, and some incurable diseases [65], SLE [66] and multiple sclerosis [67]. The MSCs perform their function in tissue microenvironment over paracrine immunoregulatory interactions with CD4+ T lymphocytes [68].

There is reported superiority in using MSC therapy in comparison with other treatments [69]. On the other hand, this idea has not been accepted by the scientific community due to some concerns of lack of adequate evidence. Published evidence suggests that MSCs-based treatment, though, have some advantages along with some drawbacks which are illustrated in Table 7. Apart from these, there are certain bottlenecks in the research of the stem cell therapy concept that need to be overcome; this important topic will be the focus of another article.

Case reports of the SC therapy in COVID-19 disease is contributing a lot to the scientific evidence and shedding light to the clinical trial hypotheses. In the recent one, a severely ill COVID-19 patient with brain and multi-organ impairment was treated with WJ-MSCs, when the patient has not demonstrated any improvement with the conventional treatment, WJ-MSCs were transplanted to the patient four times intravenously. The authors reported that there were no side effects observed in the patient. Alleviation of the cytokine storm, improved pulmonary function accompanied by the improved other organ systems such as brain, opened the road of discharge of the patient with recovery and no adverse events due to the SC treatment [16].

**Table 7:** The Pros and Cons of MSCs use for treatment purposes in general*.

PROS	Comments on PROS	CONS	Comments on CONS
Accessibility	They are easily accessible and can be isolated from various tissues	Cell Sourcing	The cell-based therapies need the cells need to be expanded in large quantities while protecting their uniformity in activity and staying pathogen free. The use of human embryonic stem (ES) cells versus adult SCs is still a matter of debate. The current political decision makers strongly favoring adult stem cell option.
Potency	They are multipotent stem cells	Plasticity	Adult SCs have certain plasticity to transdifferentiate from one lineage pathway to another. Besides, instead of transdifferentiating with normal diploid chromosomal numbers, SCs may fuse with tissue-specific differentiated cells causing polyploidy. MSCs differentiation was reported to lead to tissue ossiﬁcation or calciﬁcation in animal models which is also a potential risk in human use.
Volume	MSCs can easily expand to clinical volume in a suitable period of time	Safety Concerns of using transformed cells	In children with adenosine deaminase deficiency, the autologous transplantation of genetically modified hematopoietic stem cells was reported to lead to severe immunodeficiency followed by acute leukemia in some of the patients. Nerve cells implantation in Parkinson’s disease patients was reported the high rate of severe and uncontrollable dyskinetic activity as adverse effect. Also, myoblast implantation into the heart tissue showed adverse event of increased rates of cardiac arrhythmias.
Storage	MSCs can be stored for repetitive therapeutic usage	Choosing autologous or non-autologous human cells	Regardless of the chosen method, each treatment has important regulatory and practical difficulties, and logistics of delivery can be another issue, i.e. to maintain the uniformity of cells, to avoid any contamination during cell processing.
No Reported Safety issues	Clinical trials of MSCs so far haven’t shown adverse reactions to allogeneic MSC.	Maintenance of cell viability	The nutrient and oxygen delivery to the cellular implant is of vital importance for viability of the cells. It has been still in experimental stage to use the neovascular capillary bed in and around the cell implant and intravascular cell encapsulation implant approach not having use in human in clinics.
Effectiveness	Effectiveness of MSCs have been obviously documented in several clinical trials		*The references used to compile the information for this table: 22, 33, 77, 78, 79, 80, 81.

The ICTP database WHO, displays the clinical trial efforts on developing new therapy for COVID-19 disease. In 1135 trials in total dealing with COVID-19 treatment, only 16 trials have COVID-19 cure with stem cells, having defined certain clinical development phase (1,4%). Besides this relatively low interest, the development phases of the selected trials show that all the trials are in early development phases (Diagram 1). One trial is of phase 0 which is early phase 1 in current terms researching in the exploratory way; 8 trials are of phase 1 which is first in human safety studies; 4 trials are of phase 1 /2 meaning that these trials are fulfilling both phase objectives of determining early safety data along with collecting data on the efficiency of the treatment; 3 trials are in phase 2 investigating in the therapeutic confirmatory directions.

It is not surprising that there are no phase 3 trials yet, which is the step when the treatment is tested in thousands of patients across the world providing strong statistical evidence on effectivity and the safety of the treatment, in the database. This indicates that development phase progress has not reached to this level since the adequate amount of evidence to go forward has not been collected and reported so far due to at least time restrictions forced by the urgency of the cases effected by the novel coronavirus.

There are certain research tools used to minimize the research bias, including randomization, and blinding. The first published randomized controlled trial (RCT) appeared in the 1948 [70], and by the late 20th century, RCTs were recognized as the standard method for ‘rational therapeutics’ and ‘gold standard’ of evidence-based medicine [71]. When we evaluate the use of these tools in the selected trials on MSC treatment ofCOVID-19, there are 4 studies used both randomization and blinding (Table 3).

Two phase 1 / 2 studies having randomized triple blinding design of two arms. The umbilical MSCs (NCT04339660) and allogeneic human dental pulp MSCs (NCT04336254) are used, respectively. In both trials, the SC therapy is applied as add on therapy, to the conventional treatment, in a placebo controlled manner. The NCT04339660 trial is designed to provide proof for the efficiency with measures of inflammation and cytokine reactions of pulmonary healing,. Similarly, the NCT04336254 trial has the main aim to define the time to clinical improvement, and the adverse effects follow up is listed in secondary outcome measures not in primary ones.

The patient sampling size is quite small in these trials not allowing a reliable prediction for larger population. Also the primary outcome measures need to be reported in the registries when obtained and processed and need to be published so that the scientific society can syllogize the trial outcomes defined versus obtained.

Leng et. al, recently reported a pilot trial which was conducted as parallel design in seven patients with COVID-19 pneumonia treated with intravenous administration of clinical-grade human MSCs [72]. The placebo group consisted of three patients. Reportedly, the patients who are selected for their being in range of severe to critical conditions showed alleviation in all symptoms s by 2-4 days after receiving MSC infusion with no apparent adverse effects. These findings were supported by the abating pneumonia infiltration indicated in chest CT, and negative results for the SARS-CoV-2 nucleic acid test in two weeks after infusion [72]. The mechanisms underlying the MSC infusion-led improvements in COVID-19 patients attributed to strong antiinflammatory action of MSCs. The evidence of this was obtained from multiple beneficial outcomes, including the increased number of peripheral lymphocytes, the decline in the C-reactive protein, and waning of overactivated cytokine-secreting immune cells (CD4+ T cells, CD8+ T cells, and NK cells) by 3-6 days in the circulating blood [72, 73]. The improvement was found exciting, especially in elderly patient, which provided preliminary proof of efficacy and short term safety of the treatment. However, this trial has a very limited number of subjects, and the effectiveness of the treatment and adverse events follow up was done in a very short time period 2, 4, 6, 9, 14 days after treatment, not aiming to get long term effectiveness and safety data over long term study outcome measures.

This analysis is required further validation with a systemmatical approach of published and nonpublished clinical trials data with certain selection criteria.The treatment with MSCs of patients with COVID-19 pneumonia was found safe and effective, especially critically severe cases, as reported by an investigator based on some review on clinical trials [74]. In a phase I, multi-center, open label, dose escalation study, [64] nine patients with moderate to severe ARDS were enrolled. The patients received a single intravenous allogeneic BM-MSCs infusion with low (1x10^6^ MSCs/kg), intermediate (5x10^6^ MSCs/kg) or high dose (10x106 MSCs/kg). No treatment-related clinical instability, adverse events or toxicity was reported at the doses tested. High dose BM-MSCs demonstrated better sequential organ failure assessment (SOFA) score than lower doses. But there were no significant differences in ARDS markers (IL-6, IL-8, ANGPT2, and AGER) detected in the samples.

In another trial, the intravenous administration of 2×10^6^ cells/kg of allogeneic BM-MSCs was tested in two patients diagnosed with severe ARDS who did not improve with all supportive therapies. Gladsomely, along with the promising treatment safety results, the recovery from multiple organ failure and presentation of reduced epithelial apoptosis, alveolar-capillary fluid leakage, proinflammatory cytokines, miRNAs, and chemokines samples in both patients were reported (75).

In a double-blind, multi-center, randomized trial, BM-MSC treatment in ARDS was assessed [76]. A single intravenous dose of 10 × 10^6^ BM-MSCs/ kg bw was given to 40 patients with moderate to severe ARDS. A well-known pulmonary and systemic vascular injury biomarker, Angiopoietin 2, is a well-recognized mediator and biomarker of pulmonary and systemic vascular injury was chosen to track the pulmonary function improvement and a strong correlation between this biomarker and cell viability and between viability and improvement in oxygenation index. The authors claimed the effective treatment in recovering patients in MSC group with no MSC-related hemodynamic or respiratory adverse events. The authors concluded that a single dose of intravenous BM-MSCs was safe in patients with moderate to severe ARDS and larger trials were needed to assess efficacy.

Hence, although the efficiency of the MSCs treatment offers promising evidence, there is still a gap in safety validation of the MSC therapy leading the scientists to be cautious in accepting the findings with inner peace. This explains why in nine out of sixteen trials in this paper have both efficacy and safety as the main objectives and primary outcome measures (ChiCTR2000029569, ChiCTR2000030138, ChiCTR2000029990, ChiCTR200 0031430, NCT04252118, NCT04276987, NCT04331 613, NCT04336254, NCT04288102). Given the fact that these trials are of early development phases, and have relatively small patient population, these trials’ results, study reports and more importantly the publications are awaited to get the insights about the outcome measures and how these provide proof particularly for safety profile.

The cancelled study during this paper’s preparation (ChiCTR2000030300) has no reason for cancellation in the registry, which needs to be another transparent data for all interested parties. More interestingly, the remaining six studies have objective and outcome measures of efficiency of the treatment, but not safety. Further study designing especially on effective treatment regimen, as well as validated safety evidence in long period of time will be enlightening the gaps.

In the trials selected in this analysis, the dose optimization and the treatment intervals are being tested mainly as conventional treatment plus UC-MSCs as 4×10^7^/kg (ChiCTR2000031430), MSCs 3×10^7^ /kg (NCT04252118), MSCs 1x10^6^ cells/kg (NCT04339660) DP-MSCs 3×10^7^ /kg (NCT04336254) WJ-MSCs 3X10^6^/kg (NCT04313322 and NCT04313322), MSCs as 2x10^7^ cells (NCT04315987) in route of intravenous infusion. MSCs-derived exosomes (NCT04252118) are tested over inhalation in 2.0x10^8^ nano vesicles/3 ml. As seen, the doses of MSC infusions are still in experimental stage. Moreover, in trial NCT04331613, the design consisted of 3 cohorts with 3 patients per cohort, the dose is defined as 3, 5 or 10 million cells/kg; if there are no safety concerns for each cohort, the dose is planned to be escalated from lower dose to next higher dose.

Treatment intervals also seems a matter of investigation, since some trials select once in three days infusion for a total of three times, whereas some others prefer to give the infusion once in two days, following up the patients 21 or 28 days. On the other hand, in 5 trials both dose of the cell-based treatment and the treatment intervals were not mentioned in the registry, there are also some trials did not provide the target COVID-19 disease severity information, whereas some others mention that severe or critically ill cases as the target of the treatment (Table 6). In order to meet the trial outcome targets, the trials need to be evaluated with disease severity levels, the doses infused to the patients and the expected pulmonary improvement and safety indicators.

We consider this picture in a couple of ways: Firstly, the trial registry has not been used by all responsible parties to allow society to understand the doses and dosing intervals definition as we are missing information, like dose and treatment interval details, to get a general idea on dosing strategy. Secondly, used dosing regimens, dosing intervals and follow up durations vary among the trials, probably due to the experimental nature, sources, clinical-stage of the dose context of the MSCs. When the information on the severity of the COVID-19 target cases is not given in trial information, then the validity of the data needs to be justified since the disease is appearing in varying severities due to the individual differences [7, 77].

The cytokine storm and the relevant pulmonary and/or other tissue damages could vary depending on the age and the concomitant other chronic diseases [78, 79, 80, 81]. As the ages of the included patients almost at all selected trials, there is one more concern raise: Can the same dosing strategy be followed confidently at all ages between 18 and 76 (note: average of inclusion age maximum at all trials, except the ones that maximum age was mentioned as ‘older’ only).

In this paper, reviewing the published information and clinical trials’ data in WHO registry we suggest the following:

1 -The pilot studies bring the promising cure option for COVID-19 cases with MSC therapy as add-on treatment into scientific society’s attention.
2 -Early-stage clinical trials have demonstrated and keep focusing on the efficiency and safety of MSC treatment in COVID-19 patients. There is an urgent need for large-scale investigations to confirm and validate the safety and efficacy profile of these therapies with reliable evidence.
3 -There is a need for ‘reloaded’ trial designs considering the randomization and blinding along with controlled setting for the MSCs treatment investigation in larger populations across the world taking the power of the evidence into account.
4 -The registry of WHO need to be kept up-to-date with full trial design, protocol summary and also for progress of the trials for the sake of transparency in clinical trials’ data for all parties.
